# Evaluating current acute aortic syndrome pathways: Collaborative Acute Aortic Syndrome Project (CAASP)

**DOI:** 10.1093/bjsopen/zrae096

**Published:** 2024-09-19

**Authors:** Jim Zhong, Aminder A Singh, Nawaz Z Safdar, Sandip Nandhra, Ganesh Vigneswaran, CAASP Collaborators, Ishtiaq Aziz, Aseel Abuduruk, Adel Abdallah, Philip Stather, Enrico Mancuso, Ayman Elsayed, Tom Wallace, Ryan Laloo, Steve Tang, Kelsey Aimar, Ahmed Al Aufi, Jim Zhong, Choong Poon, Jun-Li Tham, Weii Jsim Leong, Ahmed Khalil, Mohamed Taha, Bharadhwaj Ravindhran, Chukwuemeka Igwe, Elio Plevneshi, Mustafa Altarqane, David C Bosanquet, Khaing Khant Oo, Brenig Gwilym, Syfe Azooz, Ayako Niina, Indrajeet Mandal, Abin Varghese, Michael Stephanou, Ehsan Ghaffari, Raman Uberoi, Katrina Harborne, Samuel Walker, Faizan Nasir, Niamh Horne, Benyamin Alam, Andrew Christie, Linda Watkins, Rebecca Cuthbertson, Lucia Lenart, Neera Chaudhary, Alisa Khalid, Ragai R Makar, Andrew I Khallaf, Chris T Francis, Shady Zaki, Ramez Shehata, Anwar Al-Kassar, Liam Musto, Nikesh Dattani, Gabriel Lopez-Penna, John S M Houghton, Mathew Pettit, James Budge, William Selway, Barnaby Farquharson, Mital Desai, Jack Mcalinden, Kriti Tripathi, Isaac Kobe, Ramesh Kannan, Charlotte Slee, Reema Munshi, Sriram Kumar Boppana, Sai Wunnava, Michael Woodmass, Caitlin Nessa Griffith, Alexandra Rachel Beth Dalton, Yousef Shahin, Victoria Burrows, Saima Ehsan, Joe Kang, Joni Tan, Thomas Geh, Drew Maclean, Sohini Chatterjee, Bukola Ogunjinmi, Marina Pearson, Deevia Kotecha, Nikesh Dattani, Ruth Benson, Robert Blair, Shian Patel, Joseph Shalhoub, Hunain Shiwani, Avik Som, Ginny Sun, Manar Khashram, Graham Cooper, Julie Sanders, Philip Scott, Sarah Wilson, Robin Williams, Paul Walker, Phil Jackson, Mathew Bromley, Graeme Ambler, Panagiota Birmpili, Louise Hitchman, Katherine Hurndall, Sarah Onida, Athanasios Saratzis, Nina Al-Saadi, Lauren Shelmerdine

**Affiliations:** Department of Diagnostic and Interventional Radiology, Leeds Teaching Hospitals NHS Trust, Leeds, UK; School of Medicine, University of Leeds, Leeds, UK; Cambridge Vascular Unit, Cambridge University Hospitals NHS Trust, Cambridge, UK; Cambridge Stem Cell Institute, University of Cambridge, Cambridge, UK; School of Medicine, University of Leeds, Leeds, UK; Department of Internal Medicine, St. James’s University Hospital, Leeds Teaching Hospitals NHS Trust, Leeds, UK; Northern Vascular Centre, Freeman Hospital, Newcastle upon Tyne Hospitals NHS Trust, Newcastle, UK; Faculty of Medicine, University of Southampton, Southampton, UK

## Abstract

**Background:**

Diagnosis of acute aortic syndrome is challenging and associated with high perihospital mortality rates. The study aim was to evaluate current pathways and understand the chronology of acute aortic syndrome patient care.

**Method:**

Consecutive patients with acute aortic syndrome imaging diagnosis between 1 January 2018 and 1 June 2021 were identified using a predetermined search strategy and followed up for 6 months through retrospective case note review. The UK National Interventional Radiology Trainee Research and Vascular and Endovascular Research Network co-ordinated the study.

**Results:**

From 15 UK sites, 620 patients were enrolled. The median age was 67 (range 25–98) years, 62.0% were male and 92.9% Caucasian. Type-A dissection (41.8%) was most common, followed by type-B (34.5%); 41.2% had complicated acute aortic syndrome. Mode of presentation included emergency ambulance (80.2%), self-presentation (16.2%), and primary care referral (3.6%). Time (median (i.q.r.)) to hospital presentation was 3.1 (1.8–8.6) h and decreased by sudden onset chest pain but increased with migratory pain or hypertension. Time from hospital presentation to imaging diagnosis was 3.2 (1.3–6.5) h and increased by family history of aortic disease and decreased by concurrent ischaemic limb. Time from diagnosis to treatment was 2 (1.0–4.3) h with interhospital transfer causing delay. Management included conservative (60.2%), open surgery (32.2%), endovascular (4.8%), hybrid (1.4%) and palliative (1.4%). Factors associated with a higher mortality rate at 30 days and 6 months were acute aortic syndrome type, complicated disease, no critical care admission and age more than 70 years (*P* < 0.05).

**Conclusions:**

This study presents a longitudinal data set linking time-based delays to diagnosis and treatment with clinical outcomes. It can be used to prioritize research strategies to streamline patient care.

## Teaser

The Collaborative Acute Aortic Syndrome Project is a multicentre UK service evaluation presenting a longitudinal data set linking time-based delays to diagnosis and treatment with clinical outcomes.

## Introduction

Acute aortic syndrome (AAS) encompasses a spectrum of life-threatening emergency aortic conditions including aortic dissection (Ad), intramural haematoma (IMH) and penetrating atherosclerotic ulcer (PAU), with dissection accounting for over 90% of cases^1^. AAS has an estimated population incidence of 2–16 per 100 000 persons and outcomes remain poor with high perihospital mortality rates^2–4^. Multiple risk factors exist for AAS including those that increase intimal shear stress, for example hypertension, and those that weaken the vessel wall, for example atherosclerosis or connective tissue disorders^5^. Some patients also have a family history of AAS, with 13–22% having an affected first-degree relative^5^.

There are approximately 2500 cases per year in England, although some have suggested that the condition is under-reported in part due to its varied clinical presentations^6,7^. Symptoms include chest and back pain that can mimic a range of other more common conditions with successful diagnosis reliant on a high degree of clinical suspicion alongside a low threshold for cross-sectional imaging. Early recognition is key to the successful management of patients with acute Ad, however, in 16–40% of cases there is a delay in diagnosis that can lead to an adverse outcome^7^.

At present there is limited understanding of the current pathways used to diagnose AAS. Clinical ‘red-flag’ symptoms, such as truncal pain and syncope, have historically demonstrated limited accuracy^8^. Although the literature suggests that one of the more sensitive (62–78%) symptoms for AAS is truncal pain, it is a very common presenting complaint and may represent several other differential diagnoses^9^. A previous study identified several clinical factors that were associated with delays in diagnosis such as female sex, age more than 70 years, diabetes mellitus and painless presentation, however, these studies have not been validated^10^. Identifying the interval(s) within a patient’s pathway where delay occurs, such as between symptom onset and admission, or time to imaging or treatment, remains challenging to elucidate. Retrospective studies are limited by data availability and prospective studies struggle to recruit suitable numbers of positive AAS patients given the relatively low incidence in comparison to similar presenting pathologies such as myocardial infarction^11^.

Significant variation exists in AAS diagnostic pathways across regions, with many emergency departments not having a formal work-up pathway for AAS^2^. This likely affects the time to diagnosing AAS, which is time-sensitive given the need for urgent management and potential intervention. These diagnostic delays could impact patient outcomes.

The Collaborative Acute Aortic Syndrome Project (CAASP) was developed with patients to evaluate current pathways and understand chronology of AAS patient care. Further aims were to highlight clinical, diagnostic and management factors that might impact on clinical outcomes through time-based delays.

## Methods

### Project design

This was a multicentre, retrospective service evaluation pertaining to adult patients diagnosed on imaging with AAS. All hospitals with an accident and emergency unit were eligible to participate. The study was co-ordinated and delivered through collaboration between the UK National Interventional Radiology Trainee Research (UNITE) network and the Vascular and Endovascular Research Network (VERN). Open invitations to participate were circulated via social media, national radiology and vascular surgery society mailing lists, and personal communications. The study protocol has been published^12^.

### Project approvals

CAASP was approved as a national service evaluation project by the lead organization Leeds Teaching Hospitals NHS Trust (approval date 18 May 2022, ref: CAASP). All participating UK sites were required to locally register the study as a national service evaluation project before data collection. Ethical approval was not required in the UK as no patient identifiable information was collected and there was no impact on routine care.

### Participants

All adult patients (age more than 18 years) with a diagnosis of AAS (type A dissection, type B dissection, non-A non-B dissection, PAU, IMH) on imaging between 1 January 2018 and 1 June 2021 were included. Exclusion criteria were non-AAS pathology (for example traumatic or iatrogenic aortic injury) or chronic presentation of aortic disease.

AAS was defined using the European Society of Cardiology guideline^13^. Complicated AAS was defined as the presence of end organ malperfusion or aortic rupture. AAS patients were identified at each enrolled centre (by the local study team) using a focused search strategy on electronic radiology information systems (*Appendix S2*). Data were retrospectively collected for AAS patients diagnosed between 1 January 2018 and 1 June 2021 using a template data form. Follow-up data was collected for a duration of 6 months from the first imaging diagnosis of AAS. The start date for centres was variable to allow for local registration and approval, however, the end date for centres submitting data was 31 March 2023. AAS patients were cross-checked against prospectively maintained local databases of AAS patients to ensure identification of all possible patients.

### Project outcomes

The primary aim was to capture information on the time taken for patients with AAS to attend hospital from the onset of symptoms, the time from hospital presentation to imaging diagnosis and time from imaging diagnosis to management/treatment instigation.

The primary endpoint was to determine the impact of the total and individual time components on the patient mortality rate within 30 days and 6 months. Secondary endpoints included additional clinical (for example presentation symptoms, signs, observations, vital signs, co-morbidities and blood test results) and demographic (for example age, sex, ethnicity, distance to hospital, deprivation score) variables correlated with time to hospital presentation, diagnosis and treatment and subsequent mortality rate outcomes. The coronavirus disease (COVID)-19 group included all patients who presented on or after 23 March 2020, when the UK national lockdown started. Before this date, the patients were labelled as a ‘Non-COVID’ group to investigate if this was a significant variable affecting the study outcomes.

### Sample size

This project was designed to evaluate current AAS pathways, therefore, no minimum sample size was required to show effect. Centres with fewer than five patients were excluded.

### Data management

Deidentified data were transferred to a UK NHS server (based at Leeds Teaching Hospitals NHS Trust, Leeds) as per NHS and Caldicott Guardian principles. Data sharing agreements were adhered to. Each centre was required to record local identifiers on a secure, local, and general data protection regulation compliant database to allow data capture and linkage.

### Data analysis

Summary statistics regarding time to presentation, time to imaging diagnosis, time to treatment, mortality rate and complications for AAS patients within the first 6 months of imaging diagnosis are presented. Time outcomes are presented as median time (i.q.r.).

Factors associated with both mortality rate and delay were calculated using multivariable analysis. The choice of variables to include in this model was based on consensus opinion from the steering group. A multiway analysis of variance (ANOVA) was used to calculate individual factors that contributed to timepoint measures, with handling of continuous variables (for example age and distance) with analysis of covariance (ANCOVA). Extreme outliers were examined by plotting raw data and removed if data was greater than the 99th centile. Statistical significance was measured at *P* < 0.050, with post hoc comparisons by Tukey–Kramer. Geometric means, approximate standard deviations and 95% confidence intervals (95% c.i.) are reported for the significant variables by back-transformation to allow for an understanding of reported significant variables rather than reporting normalized log transformed means. Differences in diagnostic pathways between specialist cardiovascular centres and non-cardiovascular centres, geographical and socioeconomic (using the index of multiple deprivation scores) variation were assessed to provide an insight into the variability of management.

Binomial logistic regression was used to identify factors that were significantly associated with survival metrics (alive at 30 days and 6 months). Significant factors were then used in a multivariable Cox proportional hazards model. Age was dichotomized around 70 years, all timepoints (for example time from symptom onset to presentation) were binarized about the median. The study ‘end date’ was 31 March 2023 with all sites having to submit data by this date to be included in analyses. Those alive at this date were censored.

### Handling missing data

Missing data was highlighted and the number and percentage of individuals in the missing category are presented.

## Results

During the study interval, 620 patients from 15 UK sites were enrolled. Baseline demographics are presented in *Table 1*, clinical presentation symptoms in *Table 2* and past medical history factors in *Table 3*. Median age (range) was 67 (25–98) years, 62.0% (382 of 620) were male and 92.9% (499 of 537) were Caucasian. The most frequent AAS was type-A dissection (41.8%), followed by type-B (34.5%), IMH (12.1%), PAU (9.4%) and non-A non-B dissection (2.3%), while 41.2% had complicated AAS. The most common presenting symptom was sudden onset pain (79.9%). Mode of presentation included by emergency ambulance transfer (80.2%), self-presentation (16.2%) and primary care referral (3.6%); 60.2% were managed conservatively, 32.2% had open surgery, 4.8% had endovascular aortic repair, 1.4% had a hybrid procedure and 1.4% had palliative management.

**Table 1:** Demographics of study cohort

Demographics	Total	Predictors of time from symptom onset to hospital presentation *P*	Predictors of time from hospital presentation to imaging diagnosis *P*	Predictors of time from imaging diagnosis to treatment *P*
Age (years), median (range), (*n* = 620)	67 (25–98)	0.459	0.988	0.918
**Centre type (*n* = 620)**		0.802	0.665	0.102
DGH	45 (7)			
Tertiary	575 (93)			
**Sex (*n* = 620)**		0.385	0.756	0.589
Male	382 (62)			
Female	238 (38)			
**Ethnicity (*n* = 537)**		0.887	0.410	0.249
Caucasian	499 (92.9)			
Black	17 (3.2)			
Asian	17 (3.2)			
Other	4 (0.7)			
**Index of multiple deprivation decile**		0.348	0.707	0.594
1 (most deprived)	68 (11.5)			
2	42 (7.1)			
3	52 (8.8)			
4	54 (9.2)			
5	54 (9.2)			
6	72 (12.2)			
7	66 (11.2)			
8	57 (9.7)			
9	55 (9.3)			
10 (least deprived)	69 (11.7)			
Distance from presenting hospital (miles), median (range)	6.4 (1–199)	0.807	N/A	0.271
**Aortic pathology (*n* = 620)**		0.664	0.282	0.059
Type A	226 (41.8)			
Type B	190 (34.5)			
Non-A non-B	13 (2.3)			
Penetrating aortic ulcer	48 (9.4)			
Intramural haemorrhage	60 (12.1)			
Complicated disease (*n* = 565)	233 (41.2)	N/A	N/A	0.495
**Mode of presentation (*n* = 561)**		0.163	0.386	N/A
Direct self-presentation	91 (16.2)			
Ambulance	450 (80.2)			
GP referral	20 (3.6)			

Values are *n* (%) unless otherwise stated. For each time outcome (symptom onset to hospital presentation, hospital presentation to imaging diagnosis and imaging diagnosis to treatment), the *P* values for the included variables are presented. DGH, district general hospital; N/A, not applicable.

**Table 2:** Clinical presentation symptoms and signs of study cohort

Presenting symptoms and signs	Total	Predictors of time from symptom onset to hospital presentation *P*	Predictors of time from hospital presentation to imaging diagnosis *P*	Predictors of time from imaging diagnosis to treatment *P*
Chest pain (*n* = 588)	430 (73.1)	0.145	0.519	0.425
*Sudden onset pain (*n* = 567)	453 (79.9)	0.0001 (↓)	0.597	0.811
Severe intensity pain (*n* = 532)	405 (76.1)	0.945	0.964	0.201
Back pain (*n* = 547)	361 (66.0)	0.619	0.125	0.376
Abdominal pain (*n* = 526)	194 (36.9)	0.869	0.523	0.429
*Migratory pain (*n* = 499)	219 (43.9)	0.020 (↑)	0.165	0.916
Focal neurology (*n* = 486)	74 (15.2)	0.115	0.550	0.645
Collapse (*n* = 539)	107 (19.9)	0.050	0.219	0.573
New murmur (*n* = 479)	42 (8.8)	0.963	0.173	N/A
Ischaemic limb (*n* = 511)	50 (9.8)	N/A	0.028	N/A
Hypotension (*n* = 586)	121 (20.7)	N/A	0.914	N/A
Shock (*n* = 579)	72 (12.4)	N/A	N/A	N/A
Cardiac tamponade (*n* = 564)	43 (7.6)	N/A	N/A	N/A
Fever (*n* = 574)	16 (2.8)	N/A	N/A	N/A
Coma (*n* = 594)	74 (12.5)	N/A	0.090	N/A

Values are *n* (%). For each time outcome (symptom onset to hospital presentation, hospital presentation to imaging diagnosis and imaging diagnosis to treatment), the *P* values for the included variables are presented and significant variables highlighted with *. ↓ indicates significant decrease in time from symptom onset to hospital presentation and ↑ indicates significant increase in time from symptom onset to hospital presentation. N/A, not available.

**Table 3:** Co-morbidities and past medical history details of study cohort

Co-morbidities/past medical history	Total	Predictors of time from symptom onset to hospital presentation *P*	Predictors of time from hospital presentation to imaging diagnosis *P*	Predictors of time from imaging diagnosis to treatment *P*
*Hypertension (*n* = 577)	338 (58.6)	0.025 (↑)	0.847	N/A
Known aortic aneurysm (*n* = 568)	88 (15.49)	0.481	0.509	N/A
Previous aortic dissection (*n* = 562)	25 (4.5)	0.919	0.930	N/A
Bicuspid aortic valve (*n* = 480)	7 (1.5)	0.618	0.453	N/A
History of myocardial infarction (*n* = 561)	71 (12.7)	0.617	0.218	N/A
Cardiac failure (*n* = 565)	38 (6.7)	0.566	N/A	N/A
Previous cardiac surgery (*n* = 562)	50 (8.9)	0.458	N/A	N/A
Prior cardiac catheterization/angioplasty (*n* = 548)	44 (8.0)	0.808	N/A	N/A
*Familial aortic disease (*n* = 350)	19 (5.4)	0.320	0.019 (↑)	N/A
Known Marfan or connective tissue disease (*n* = 487)	23 (4.7)	0.286	0.775	N/A
Diabetes (*n* = 568)	66 (11.6)	0.489	N/A	N/A
**Treatment (*n* = 580)**		N/A	N/A	0.057
Medical	349 (60.2)			
Surgery	187 (32.2)			
Endovascular	28 (4.8)			
Hybrid	8 (1.4)			
Palliative	8 (1.4)			
*Transfer to another centre (*n* = 606)	181 (29.9)	N/A	N/A	0.012 (↑)
Admission to critical care (*n* = 558)	436 (78.1)	N/A	N/A	N/A
Presented during COVID (23 March 2020–1 June 2021)	240 (38.7)	0.589	0.397	0.803
Hb (*n* = 605), median (range)	133 (49–199)	N/A	0.606	N/A
Creatinine (*n* = 609), median (range)	87 (28–627)	N/A	0.593	N/A
First recorded systolic BP (*n* = 569), median (range)	141 (49–287)	N/A	0.751	N/A
First recorded diastolic BP (*n* = 561), median (range)	79 (23–190)	N/A	0.812	N/A
First recorded HR (*n* = 569), median (range)	75 (30–162)	N/A	0.661	N/A
First recorded RR (*n* = 539), median (range)	18 (10–96)	N/A	0.299	N/A

Values are *n* (%) unless otherwise stated. For each time outcome (symptom onset to hospital presentation, hospital presentation to imaging diagnosis and imaging diagnosis to treatment), the *P* values for the included variables are presented and significant variables highlighted with *. ↑ indicates significant increase in time. N/A, not available; Hb, haemoglobin; BP, blood pressure; HR, heart rate; RR, respiratory rate.

### AAS factors associated with time

**Fig. 1:**
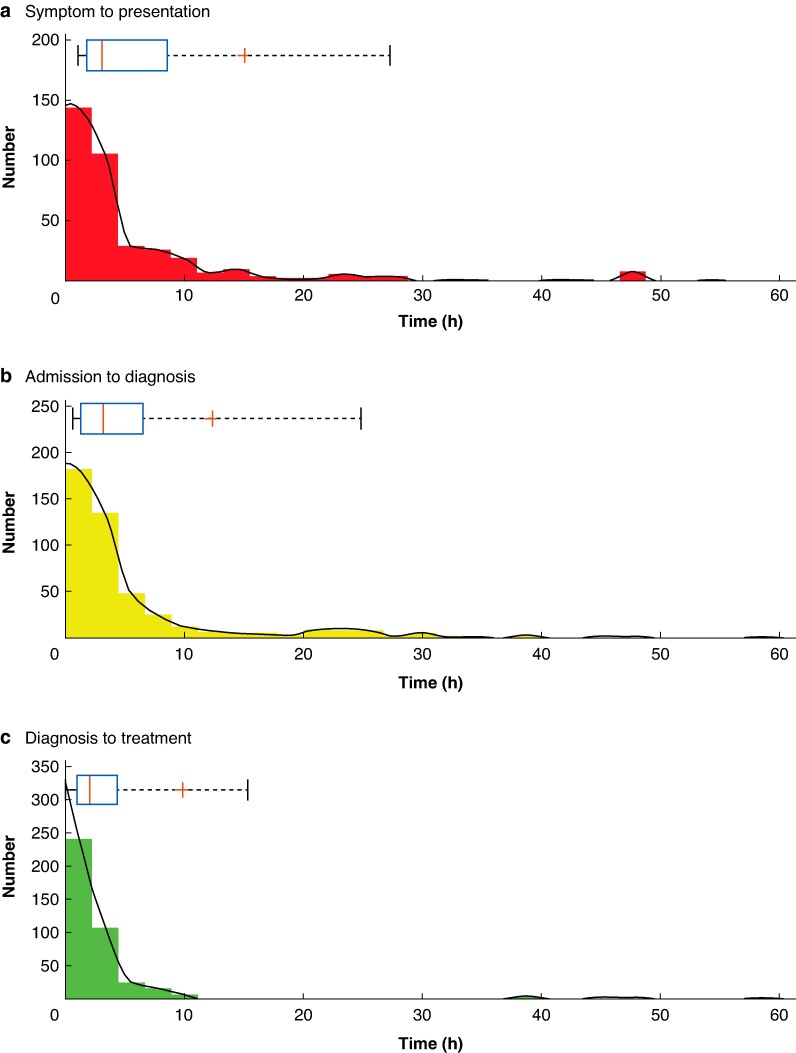
Histograms demonstrating the distribution for times from symptom onset to hospital presentation a, admission to imaging diagnosis b and diagnosis to treatment c Smoothed data is plotted in black. Above individual histograms are box and whisker plots with a red line for median, ‘+’ for the mean, a blue box around the 25 and 75% quartiles and whiskers bounding 9 and 91%.

**Fig. 2:**
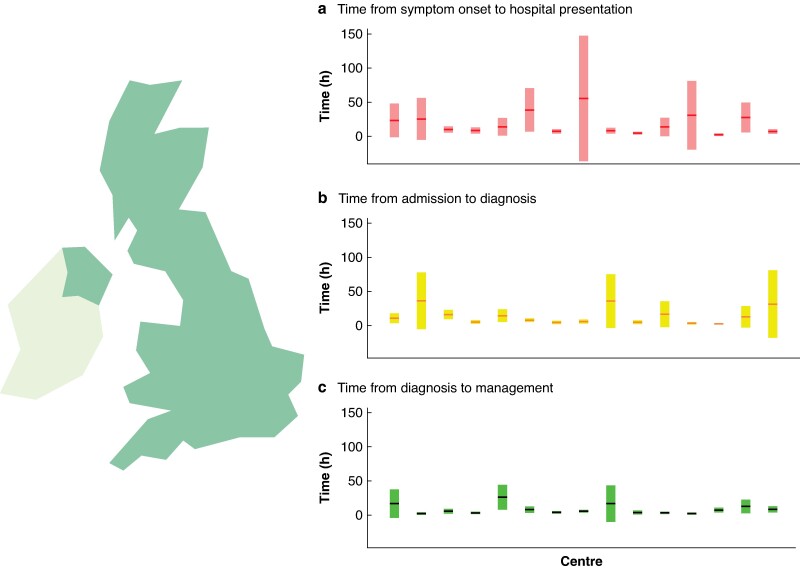
Distribution of timepoints over the 15 UK centres Mean time points for each centre are shown as horizontal lines and are placed over a 1.96× standard error of the mean (95% confidence interval) in **a** time from symptom onset to hospital presentation, **b** time from admission to imaging diagnosis and **c** time from diagnosis to treatment, expressed in hours.

The median time (i.q.r.) from symptom onset to hospital presentation was 3.1 (1.8–8.6) h, time from hospital presentation to imaging diagnosis was 3.2 (1.3–6.5) h and from imaging to initial treatment was 2.0 (1.0–4.3) h. The distribution of the three outcome timepoints is shown in *Fig. 1*. Deidentified individual centre data are shown in *Fig. 2*, highlighting the distribution of time points over the 15 UK centres.

A history of hypertension, sudden onset chest pain or migratory pain was significantly associated with the time from symptom onset to hospital presentation. Patients with hypertension had significantly longer times to hospital presentation (geometric mean 4.8 h (95% c.i. 4.1 to 5.6)) than those without a history of hypertension (geometric mean 4.5 h (95% c.i. 3.7 to 5.5)). Patients with sudden onset pain had significantly shorter times to hospital presentation (geometric mean = 3.8 h (95% c.i. 3.4 to 4.3)) than those without (11.6 h (95% c.i. 7.5 to 17.6)). Patients with migratory pain (219 of 499) had slightly longer times to hospital presentation (geometric mean = 4.8 h (95% c.i. 3.9 to 5.7)) than those without migratory pain (4.4 h (95% c.i. 3.7 to 5.3)).

A family history of aortic disease (*P* < 0.01) or concurrent ischaemic limb (*P* = 0.028) significantly impacted upon the time from hospital presentation to imaging diagnosis. A family history of aortic disease was associated with a longer time to diagnosis (5.7 h (95% c.i. 3.0 to 10.3) *versus* 3.7 h (95% c.i. 3.2 to 4.3)). Patients with concurrent ischaemic limb had much shorter times to diagnosis compared with those without (1.5 h (95% c.i. 1.1 to 2.0) *versus* 4.4 h (95% c.i. 3.8 to 5.0)).

Patients who were transferred to another centre had significantly longer times from imaging diagnosis to initial treatment (3.3 h (95% c.i. 2.7 to 4.1)), compared with patients who received their care within the same hospital (2.6 h (95% c.i. 2.3 to 3.0), *P* = 0.01). Notably, the treatment administered demonstrated a trend (*P* = 0.056) for endovascular approach (9.3 h (95% c.i. 4.5 to 18.0)) leading to a longer time to initial treatment than medical/conservative management (2.7 h (95% c.i. 2.3 to 3.1)), open surgery (2.5 h, (95% c.i. 2.0 to 3.1)), hybrid (2.8 h (95% c.i. 1.2 to 5.5)) or palliative (0.5 h (95% c.i. 0.4 to 0.7)).

**Fig. 3:**
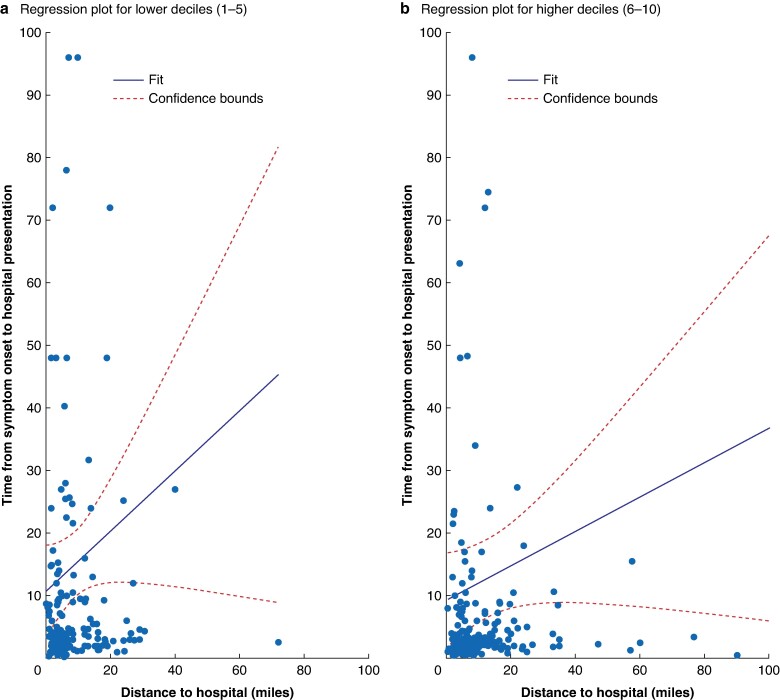
Distance from hospital and time from onset to hospital presentation stratified by index of deprivation a (low 1–5) and b high (6–10)

Distance from hospital and time from onset to hospital presentation was stratified by index of deprivation (*Fig. 3*) between low (1–5) and high (5–10) deprivation and demonstrates that those with a high index of deprivation lived a closer distance to the hospital but did not demonstrate a reciprocal decrease in time from symptom onset to hospital presentation (*P* = 0.001).

### Factors associated with mortality rate

**Fig. 4:**
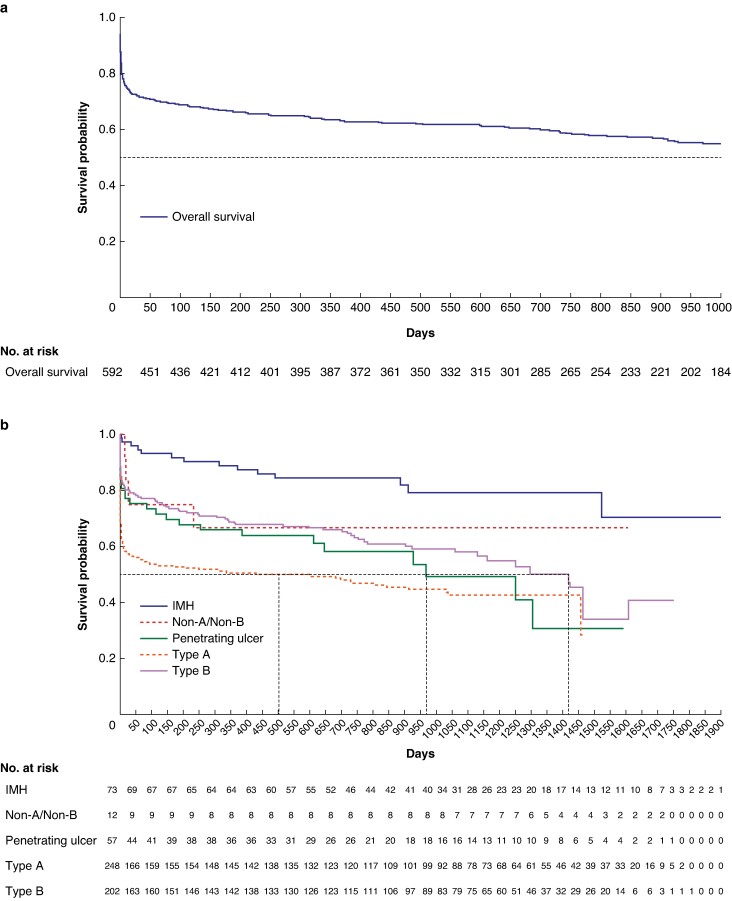

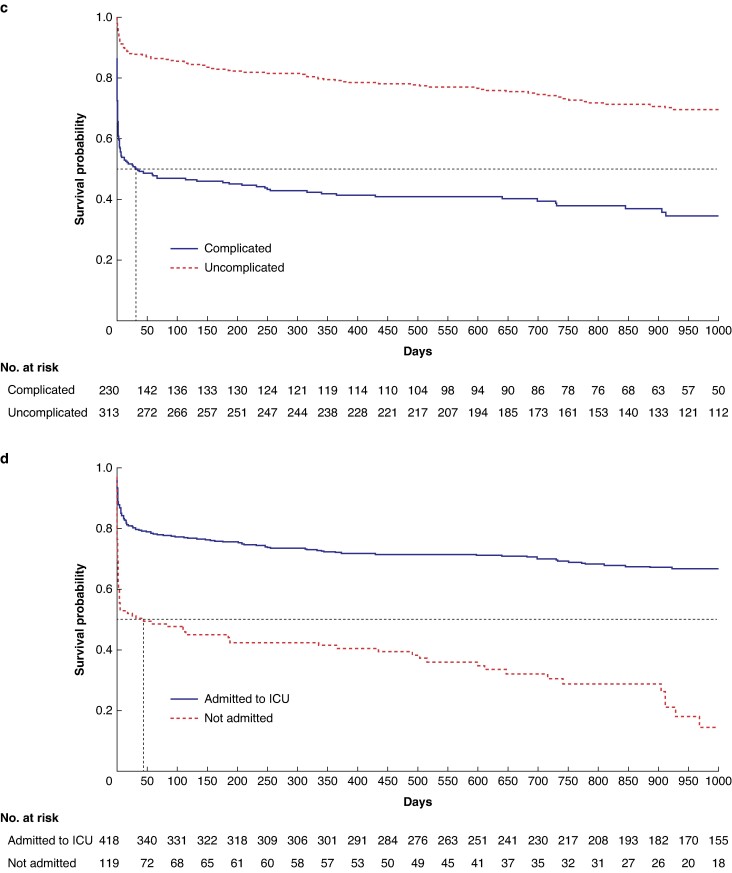

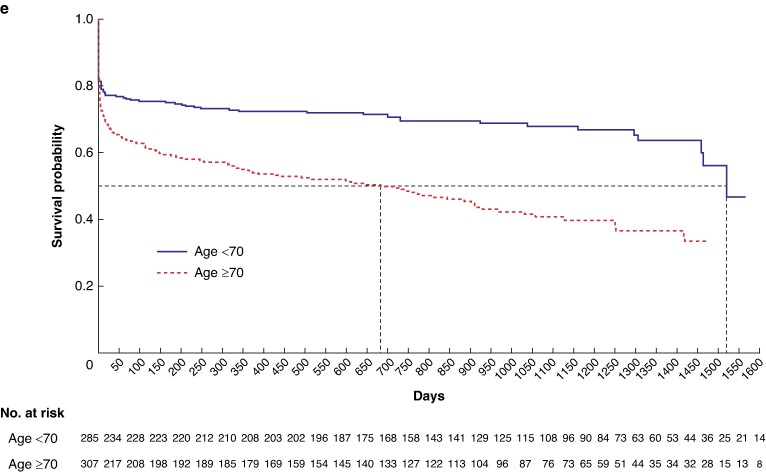
Kaplan–Meier survival curves: (a) overall; and for each of the independent predictors of mortality rate including: (b) aortic pathology, (c) presence of complicated disease, (d) admission to critical care and (e) age less than 70 years IMH, intramural haematoma.

Predictors of 30-day and 6-month mortality rates are demonstrated in *Table 4* with *Fig. 4* showing statistical analysis of these variables. A multivariable Cox proportional hazards model was used to investigate whether age, type of AAS, presence of complicated disease, admission to critical care, time from symptom onset to hospital presentation, time from hospital presentation to diagnosis, time from diagnosis to treatment and whether the AAS occurred during COVID-19, were significantly associated with survival. Age more than 70 years (HR 0.51 (95% c.i. 0.37 to 0.70), *P* < 0.001), aortic pathology (HR 0.80 (95% c.i. 0.70 to 0.90), *P* < 0.001) (see *Fig. 4* for breakdown of categories), complicated disease (HR 2.2 (95% c.i. 1.6 to 3.0), *P* < 0.001) and admission to critical care (HR 0.41 (95% c.i. 0.29 to 0.56), *P* < 0.001) were all significantly associated with survival. No time variables were significantly associated with survival, and there was no effect found for whether the patient was managed during the COVID-19 pandemic.

**Table 4:** Binomial logistic regression predictors of 30-day and 6-month mortality rate

Term	Odds ratio of being alive at 30 days (95% c.i.)	Alive at 30 days (*P*)	Odds ratio of being alive at 6 months (95% c.i.)	Alive at 6 months (*P*)
Time from symptom onset to hospital presentation (h)	1.01 (0.99,1.02)	0.551	0.99 (0.98,1.01)	0.450
Time from hospital presentation to imaging diagnosis (h)	1.03 (0.98,1.08)	0.214	1.0054 (0.99,1.02)	0.555
Time from imaging diagnosis to treatment (h)	0.98 (0.95,1.02)	0.408	0.99 (0.96,1.02)	0.518
Centre type	3.49 (0.17,72.08)	0.419	N/A	1.000
*Age at presentation	0.93 (0.89,0.97)	<0.001	0.93 (0.89,0.97)	<0.001
Sex	0.51 (0.19,1.36)	0.176	0.45 (0.17,1.23)	0.119
Ethnicity	1.19 (0.33,4.29)	0.787	0.87 (0.20,3.88)	0.858
Index of multiple deprivation decile	0.90 (0.77,1.05)	0.171	0.91 (0.79,1.06)	0.228
*Aortic pathology	1.65 (1.13,2.40)	0.009	1.75 (1.19,2.56)	0.004
Mode of presentation	0.68 (0.32,1.47)	0.329	0.75 (0.36,1.55)	0.436
*Complicated disease	0.19 (0.07,0.49)	<0.001	0.24 (0.09,0.62)	0.003
Treatment	1.15 (0.59,2.25)	0.685	1.10 (0.58,2.10)	0.775
History of previous aortic dissection	0.75 (0.04,13.83)	0.846	0.42 (0.02,7.22)	0.552
*Admission to critical care	3.06 (1.18,7.96)	0.022	3.21 (1.24,8.35)	0.017
COVID-19	1.50 (0.60,3.73)	0.381	1.18 (0.49,2.84)	0.705

Significant variables highlighted with *. N/A, not applicable.

## Discussion

This contemporary, multicentre data set provides a real-world evaluation of UK AAS care pathways and a longitudinal data set linking time-based delays to diagnosis and treatment with clinical outcomes. Most notable findings include time-associated predictors of symptom onset to hospital presentation, presentation to imaging diagnosis and imaging diagnosis to treatment, and significant independent predictors of mortality rate (AAS type, complicated disease, no admission to critical care and age more than 70 years).

There are several important messages from the present study. Firstly, CAASP reinforces the importance of early recognition of AAS and prompt referral to secondary care. Before the COVID-19 pandemic, an investigation by the Healthcare Safety Investigation Branch^7^ reported that approximately 20% of patients with thoracic aortic aneurysm and dissection (TAAD) die before reaching any hospital and, alarmingly, half die before admission to a specialist centre, with an estimated number of 2500 cases per year in England. The study found that diagnostic delays occurred in up to 40% of patients, particularly in those who presented to hospital themselves or if the doctors initially suspected a cardiac cause of chest pain. CAASP suggests that mode of presentation and increasing time to hospital presentation may be additional factors as patients not presenting by emergency ambulance faced greater delay.

Interestingly, patients with lower socioeconomic status lived closer to hospitals but did not demonstrate a reciprocal decrease in time to hospital presentation. This is an important finding suggesting disparity in presentation to hospital is disproportionately impacting those of lower socioeconomic status, demonstrating inequality in healthcare, and is in keeping with the published literature^14^. Educational initiatives targeting this group of patients may represent one method for improving the time taken for patients to present to hospital.

As part of the International Registry of Acute Aortic Dissection (IRAD), an analysis was undertaken to identify variables associated with delays in diagnosis and treatment in TAAD patients^10^. They found delays in diagnosis to occur more frequently in those with atypical symptoms (such as non-abrupt symptoms and when patients did not present with chest, back or any pain) and those presenting to a non-tertiary centre. Delay in time from diagnosis to surgery in IRAD was associated with a history of previous cardiac surgery, presenting without sudden onset pain and initial presentation to a non-tertiary centre. CAASP identified the presence of migratory pain or hypertension as significant predictors of delay in the time from symptom onset to hospital presentation. A meta-analysis of the Japan-Specific Health Checkups and UK Biobank^15^ suggested that acute aortic dissection events and mortality rate are significantly higher in those with hypertension, which challenges its exclusion from validated risk prediction tools such as the Aortic Dissection Detection Risk Score. Furthermore, chronic hypertension leads to molecular changes within the aortic wall such as elastic fibre loss^16^. Atypical factors should be considered when developing new educational and training materials as well as diagnostic pathways. Furthermore, abdominal pain was noted in 34% of patients, suggesting that atypical symptoms need to be routinely reviewed as part of a systems enquiry. This is particularly the case in the setting of aortic dissection where concurrent abdominal pain may indicate abdominal aortic and mesenteric vessel involvement^17^.

IRAD also demonstrated increased time from presentation to diagnosis in those of non-white ethnicity and those with a history of coronary artery bypass, ultimately resulting in an operative delay^10^. In addition, CAASP demonstrated that a requirement for transfer to a specialist unit for initial treatment after imaging diagnosis was significantly associated with delay. This suggests differential access to treatments for patients is dependent on their access to specialist services, which can impact time to treatment. Such access issues need to be addressed by policymakers through robust referral and transfer pathways to ensure AAS patients do not face a ‘postcode lottery’ when it comes to access to definitive treatment. Although not statistically significant there was a trend for delay in management of patients with type B dissection requiring either hybrid or endovascular therapy. This is not surprising as these patients often require an interval of re-evaluation after best medical therapy. This contrasts with type A dissection where the accepted treatment is to proceed with urgent open surgery.

Limb ischaemia can increase the index of suspicion of AAS and urgency of imaging, which can in part explain its significance in reducing the time delay to imaging diagnosis. Conversely, a family history of aortic disease increased time to imaging. Family history suggests a genetic component, increasing the likelihood of familial aortic disease as a differential^18^. This finding is surprising given it is standard enquiry during clinical consultation and part of the assessment in patients presenting with sudden onset chest or back pain and may be due to the small cohort of patients with family history. This challenges the assumption that the presence of a positive family history of aortic disease would lead to a higher index of suspicion of AAS and suggests more work is required in raising awareness of AAS amongst clinicians.

CAASP demonstrated significantly better survival in the patient cohort admitted to a critical care setting. Guidelines recommend aggressive management of blood pressure, heart rate and pain^18^, and the optimal setting for this is an intensive care unit (ICU). The factors leading to a worse mortality rate in the non-ICU cohort were not captured by this study and could have been due to patient characteristics such as multimorbidity, but this merits further investigation. Significantly worse survival outcomes for those presenting with complicated AAS are expected, as are the survival outcomes dependent on type of AAS. Both findings place CAASP in line with the published literature^19^ and as such timely referral to critical care for AAS patients is essential and should ideally occur soon after diagnosis.

The Oxford Vascular Study^20^ predicted a significant increase in annual dissection events from 3892 in 2010 to 6893 in 2050, partly due to the growing UK population where the number of those aged above 75 years is projected to double over the same interval, which is particularly concerning given that CAASP has identified age more than 70 years to be associated with a worse mortality rate. This demonstrates the urgent need to improve care and diagnostic pathways and determine the best treatments for this patient cohort.

The present study provides valuable information regarding the characteristics of patients presenting with AAS, which may reflect differences in diagnostic and treatment pathways across the UK. In 2021, a working party comprised of patients and clinicians, formed through a collaboration between the Vascular Society of Great Britain and Ireland and the James Lind Alliance, identified top research priorities in aortic disease, which included ‘What methods can be used to ensure that people with acute aortic conditions such as dissection are diagnosed quickly and treated without delay?’^21^, which formed the premise for CAASP. Further, the Acute Aortic Dissection Toolkit was launched in March 2021^22^. This sets out principles for the management of aortic dissection and aimed to provide practical advice and guidance for commissioners, service providers and clinicians to support system-wide improvement in AAS management. A key part of this work was audit and governance to allow measurement of the incidence and outcomes on a UK-wide basis. CAASP has contributed important information about variations in AAS care pathways, and patient characteristics associated with time and worse outcomes, that should be readily identified to optimize AAS pathways and can aid policy planning on where intervention to improve care quality is required.

The limitations of this work include its retrospective design and, due to several hospitals without electronic patient record systems, information being obtained from physical notes with risk of incomplete data. This contributed to missing variables in the data set, which was mitigated by excluding those patients from linked analyses. There may be geographical bias in the data depending on which centres contributed to the project, which were likely those with more staff and resources to enable them to participate, which could limit the generalizability of findings. However, the high number of centres and patients included reduces this bias. It was only possible to identify patients with AAS who had survived long enough for an imaging study to confirm their diagnosis. Patients who died of AAS before hospital presentation or before scan were not identified, which could bias the patient cohort.

CAASP presents a longitudinal data set linking time-based delays to diagnosis and treatment with clinical outcomes through a multicentre, UK collaborative research methodology. It can be used to prioritize research strategies to streamline patient care by highlighting where in the patient journey delays can occur.

## Collaborators

Ishtiaq Aziz (Norfolk & Norwich University Hospital NHS Foundation Trust, Norwich, UK), Aseel Abuduruk (Norfolk & Norwich University Hospital NHS Foundation Trust, Norwich, UK), Adel Abdallah (Norfolk & Norwich University Hospital NHS Foundation Trust, Norwich, UK), Philip Stather (Norfolk & Norwich University Hospital NHS Foundation Trust, Norwich, UK), Enrico Mancuso (Norfolk & Norwich University Hospital NHS Foundation Trust, Norwich, UK), Ayman Elsayed (Norfolk & Norwich University Hospital NHS Foundation Trust, Norwich, UK) Tom Wallace (Leeds Teaching Hospitals NHS Trust, Leeds, UK), Ryan Laloo (Leeds Teaching Hospitals NHS Trust, Leeds, UK), Steve Tang (Leeds Teaching Hospitals NHS Trust, Leeds, UK), Kelsey Aimar (Leeds Teaching Hospitals NHS Trust, Leeds, UK), Ahmed Al Aufi (Leeds Teaching Hospitals NHS Trust, Leeds, UK), Jim Zhong (Leeds Teaching Hospitals NHS Trust, Leeds, UK) Choong Poon (Wrightington, Wigan and Leigh Teaching Hospitals NHS Foundation Trust, Wigan, UK), Jun-Li Tham (Wrightington, Wigan and Leigh Teaching Hospitals NHS Foundation Trust, Wigan, UK), Weii Jsim Leong (Wrightington, Wigan and Leigh Teaching Hospitals NHS Foundation Trust, Wigan, UK), Ahmed Khalil (Wrightington, Wigan and Leigh Teaching Hospitals NHS Foundation Trust, Wigan, UK), Mohamed Taha (Wrightington, Wigan and Leigh Teaching Hospitals NHS Foundation Trust, Wigan, UK) Bharadhwaj Ravindhran (Hull Royal Infirmary, Hull, UK), Chukwuemeka Igwe (Hull Royal Infirmary, Hull, UK), Elio Plevneshi (Hull Royal Infirmary, Hull, UK), Mustafa Altarqane (Hull Royal Infirmary, Hull, UK) David C. Bosanquet (Aneurin Bevan University Health Board, Wales), Khaing Khant Oo (Aneurin Bevan University Health Board, Wales), Brenig Gwilym (Aneurin Bevan University Health Board, Wales), Syfe Azooz (Aneurin Bevan University Health Board, Wales), Ayako Niina (Aneurin Bevan University Health Board, Wales) Indrajeet Mandal (John Radcliffe Hospital, Oxford, UK), Abin Varghese (John Radcliffe Hospital, Oxford, UK), Michael Stephanou (John Radcliffe Hospital, Oxford, UK), Ehsan Ghaffari (John Radcliffe Hospital, Oxford, UK), Raman Uberoi (John Radcliffe Hospital, Oxford, UK) Katrina Harborne (University Hospitals Birmingham NHS Foundation Trust, Birmingham, UK), Samuel Walker (University Hospitals Birmingham NHS Foundation Trust, Birmingham, UK), Faizan Nasir (University Hospitals Birmingham NHS Foundation Trust, Birmingham, UK), Niamh Horne (University Hospitals Birmingham NHS Foundation Trust, Birmingham, UK), Benyamin Alam (University Hospitals Birmingham NHS Foundation Trust, Birmingham, UK) Andrew Christie (Queen Elizabeth University Hospital, Glasgow, UK), Linda Watkins (Queen Elizabeth University Hospital, Glasgow, UK), Rebecca Cuthbertson (Queen Elizabeth University Hospital, Glasgow, UK), Lucia Lenart (Queen Elizabeth University Hospital, Glasgow, UK), Neera Chaudhary (Queen Elizabeth University Hospital, Glasgow, UK), Alisa Khalid (Queen Elizabeth University Hospital, Glasgow, UK) Ragai R. Makar (Countess of Chester Hospital NHS Foundation Trust, Chester, UK), Andrew I. Khallaf (Countess of Chester Hospital NHS Foundation Trust, Chester, UK), Chris T. Francis (Countess of Chester Hospital NHS Foundation Trust, Chester, UK), Shady Zaki (Countess of Chester Hospital NHS Foundation Trust, Chester, UK), Ramez Shehata (Countess of Chester Hospital NHS Foundation Trust, Chester, UK), Anwar Al-Kassar (Countess of Chester Hospital NHS Foundation Trust, Chester, UK) Liam Musto (University Hospitals of Leicester NHS Trust, Leicester, UK), Nikesh Dattani (University Hospitals of Leicester NHS Trust, Leicester, UK), Gabriel Lopez-Penna (University Hospitals of Leicester NHS Trust, Leicester, UK), John S.M. Houghton (University Hospitals of Leicester NHS Trust, Leicester, UK) Mathew Pettit (St George‘s University Hospitals NHS Foundation Trust, London, UK), James Budge (St George‘s University Hospitals NHS Foundation Trust, London, UK), William Selway (St George‘s University Hospitals NHS Foundation Trust, London, UK), Barnaby Farquharson (St George‘s University Hospitals NHS Foundation Trust, London, UK), Mital Desai (St George‘s University Hospitals NHS Foundation Trust, London, UK), Jack Mcalinden (St George‘s University Hospitals NHS Foundation Trust, London, UK) Kriti Tripathi (Northampton General Hospital NHS Trust, Northampton, UK), Isaac Kobe (Northampton General Hospital NHS Trust, Northampton, UK), Ramesh Kannan (Northampton General Hospital NHS Trust, Northampton, UK), Charlotte Slee (Northampton General Hospital NHS Trust, Northampton, UK), Reema Munshi (Northampton General Hospital NHS Trust, Northampton, UK), Sriram Kumar Boppana (Northampton General Hospital NHS Trust, Northampton, UK) Sai Wunnava (Newcastle upon Tyne Hospitals, Newcastle, UK), Michael Woodmass (Newcastle upon Tyne Hospitals, Newcastle, UK), Caitlin Nessa Griffith (Newcastle upon Tyne Hospitals, Newcastle, UK), Alexandra Rachel Beth Dalton (Newcastle upon Tyne Hospitals, Newcastle, UK) Yousef Shahin (Sheffield Teaching Hospitals NHS Foundation Trust, Sheffield, UK), Victoria Burrows (Sheffield Teaching Hospitals NHS Foundation Trust, Sheffield, UK), Saima Ehsan (Sheffield Teaching Hospitals NHS Foundation Trust, Sheffield, UK), Joe Kang (Sheffield Teaching Hospitals NHS Foundation Trust, Sheffield, UK), Joni Tan (Sheffield Teaching Hospitals NHS Foundation Trust, Sheffield, UK), Thomas Geh (Sheffield Teaching Hospitals NHS Foundation Trust, Sheffield, UK) Drew Maclean (University Hospital Southampton, Southampton, UK), Sohini Chatterjee (University Hospital Southampton, Southampton, UK), Bukola Ogunjinmi (University Hospital Southampton, Southampton, UK), Marina Pearson (University Hospital Southampton, Southampton, UK), Deevia Kotecha (Manchester University NHS Foundation Trust, Manchester, UK), Nikesh Dattani (Leicester Vascular Institute, Glenfield Hospital, Leicester, UK), Ruth Benson (University of Birmingham Clinical Trials Unit, Birmingham, UK), Robert Blair (Belfast Health and Social Care Trust, Belfast, UK), Shian Patel (University Hospital Southampton NHS Foundation Trust, Southampton, UK), Joseph Shalhoub (Imperial College Healthcare NHS Trust, London, UK), Hunain Shiwani (Leeds Teaching Hospitals NHS Trust, Leeds, UK), Avik Som (Massachusetts General Hospital, Boston, USA), Ginny Sun (Harvard University, Cambridge, USA), Manar Khashram (Waikato District Health Board, Hamilton, New Zealand), Graham Cooper (Sheffield Teaching Hospitals, Sheffield, UK), Julie Sanders (St Bartholomew’s Hospital, London, UK), Philip Scott (University of Wales Trinity St David, Wales, Lampeter), Sarah Wilson (Wexham Park Hospital Emergency Department, Frimley Health NHS Foundation Trust, Slough, UK), Robin Williams (Newcastle upon Tyne Hospitals, Newcastle, UK), Paul Walker (Leeds Teaching Hospitals NHS Trust, Leeds, UK), Phil Jackson (Leeds Teaching Hospitals NHS Trust, Leeds, UK), Mathew Bromley (Leeds Teaching Hospitals NHS Trust, Leeds, UK), Graeme Ambler (Cambridge University Hospitals, Cambridge, UK), Panagiota Birmpili (Oxford University Hospitals NHS Foundation Trust, Oxford, UK), Louise Hitchman (Hull Royal Infirmary, Hull, UK), Katherine Hurndall (Imperial College London, London, UK), Sarah Onida (Imperial College Healthcare NHS Trust, London, UK), Athanasios Saratzis (Glenfield Hospital BHF Cardiovascular Research Facility, Leicester, UK), Nina Al-Saadi (Black Country Vascular Network, Dudley, UK), Lauren Shelmerdine (Newcastle upon Tyne Hospitals, Newcastle, UK)

## Supplementary Material

zrae096_Supplementary_Data

## Data Availability

Data availability will be considered on written request.
