# Supplementary material for: Evaluating current acute aortic syndrome pathways: Collaborative Acute Aortic Syndrome Project (CAASP)
Source: BJS Open. 2024 Sep 19;8(5):zrae096. doi: 10.1093/bjsopen/zrae096 (PMC11412149; doi:10.1093/bjsopen/zrae096)
Supplement: zrae096_Supplementary_Data [file zrae096_supplementary_data.zip › Supplementary_materials_1.docx]

**Supplemental information 1**

**CAASP Steering Committee**

Dr Jim Zhong – Clinical Research Fellow, University of Leeds/ Interventional Radiology Fellow, Leeds Teaching Hospitals NHS Trust

Dr Ganesh Vigneswaran - Academic Clinical Lecturer, University of Southampton/ Interventional Radiology Fellow, University Hospital Southampton NHS Foundation Trust

Mr Aminder Singh – Clinical Research Fellow, Cambridge University Hospitals

Dr Deevia Kotecha – Radiology Registrar, Manchester University NHS Foundation Trust

Mr Sandip Nandhra – Consultant Vascular & Endovascular Surgeon, Newcastle University

Mr Nikesh Dattani – Consultant Vascular & Endovascular Surgeon, Leicester Vascular Institute, Glenfield Hospital

Ms Ruth Benson – Academic Clinical Lecturer in Vascular Surgery, University of Birmingham Clinical Trials Unit

Mr Robert Blair – Specialist Registrar in Vascular Surgery, Belfast Health and Social Care Trust

Dr Shian Patel – Interventional Radiology Fellow, University Hospital Southampton NHS Foundation Trust/ Chair of the British Society of Interventional Radiology (BSIRT)

Mr Joseph Shalhoub - Consultant Vascular Surgeon, Imperial College Healthcare NHS Trust

Dr Hunain Shiwani - Clinical Research Fellow, UCL/ Radiology registrar, Leeds

Dr Nawaz Safdar – Leeds Teaching Hospitals NHS Trust

Dr Avik Som - Interventional and Diagnostic Radiology Residency, PGY-4, Massachusetts General Hospital, Boston, USA

Miss Ginny Sun – Medical Student, Harvard University

Mr Graham Cooper – Consultant Cardiac Surgeon, Sheffield Teaching Hospitals/ TADCT Research Committee

Professor Julie Sanders - Director Clinical Research, St Bartholomew’s Hospital (member of Cardiothoracic Interdisciplinary Research Network)

Dr Philip Scott - Programme Director, Institute of Management & Health, University of Wales Trinity St David

Dr Sarah Wilson - Emergency Medicine Consultant and Deputy Chief of Service, Wexham Park Hospital Emergency Department, Frimley Health NHS Foundation Trust

Dr Robin Williams, Consultant Interventional Radiologist, Newcastle and Chair of the BSIR Audit and Registry Committee

Dr Paul Walker, Consultant Interventional Radiologist, Leeds

Dr Phil Jackson - Consultant Anaesthetist and ICU Physician, Leeds

Dr Mathew Bromley - Consultant Anaesthetist

**The United Kingdom National Interventional Radiology Trainee Research Network (UNITE)**

J Zhong, G Vigneswaran, D Kotecha, I Mandal

**The Vascular and Endovascular Research Network**

G. Ambler, R. Benson, P. Birmpili, R. Blair, D. C. Bosanquet, N. Dattani, B. L. Gwilym, L. Hitchman, K. Hurndall, M. Machin, S. Nandhra, S. Onida, A. Saratzis, J. Shalhoub, A. A. Singh, N. Al-Saadi, L. Shelmerdine

**List of collaborators**

Norfolk & Norwich University Hospital NHS Foundation Trust

Dr Ishtiaq Aziz

Dr Aseel Abuduruk

Dr Adel Abdallah

Dr Philip Stather

Dr Enrico Mancuso

Dr Ayman Elsayed

Leeds Teaching Hospitals NHS Trust

Mr Tom Wallace

Mr Ryan Laloo

Mr Steve Tang

Dr Kelsey Aimar

Dr Ahmed Al Aufi

Dr Jim Zhong

Wrightington, Wigan and Leigh Teaching Hospitals NHS Foundation Trust

Dr Choong Poon

Dr Jun-Li Tham

Dr Weii Jsim Leong

Dr Ahmed Khalil

Dr Mohamed Taha

Hull

Dr Bharadhwaj Ravindhran

Dr Chukwuemeka Igwe

Dr Elio Plevneshi

Dr Mustafa Altarqane

Aneurin Bevan University Health Board (WALES)

Mr David Bosanquet

Mr Khaing Khant Oo

Mr Brenig Gwilym

Dr Syfe Azooz

Dr Ayako Niina

Oxford

Dr Indrajeet Mandal

Dr Abin Varghese

Dr Michael Stephanou

Dr Ehsan Ghaffari

Dr Raman Uberoi

Birmingham

Dr Katrina Harborne

Dr Samuel Walker

Dr Faizan Nasir

Dr Niamh Horne

Dr Benyamin Alam

Glasgow

Dr Andrew Christie

Dr Linda Watkins

Dr Rebecca Cuthbertson

Dr Lucia Lenart

Miss Neera Chaudhary

Alisa Khalid

Chester

Dr Ragai R. Makar

Dr Andrew I. Khallaf

Dr Chris T. Francis

Dr Shady Zaki

Dr Ramez Shehata

Dr Anwar Al-Kassar

Leicester

Mr Liam Musto

Mr Nikesh Dattani

Mr Gabriel Lopez-Penna

Mr John SM Houghton

St Georges, London

Dr Mathew Pettit

Mr James Budge

Dr William Selway

Dr Barnaby Farquharson

Mr Mital Desai

Dr Jack Mcalinden

Northampton

Dr Kriti Tripathi

Dr Isaac Kobe

Mr Ramesh Kannan

Dr Charlotte Slee

Dr Reema Munshi

Dr Sriram Kumar Boppana

Newcastle

Dr Sai Wunnava

Dr Michael Woodmass

Dr Caitlin Nessa Griffith

Dr Alexandra Rachel Beth Dalton

Sheffield

Dr Yousef Shahin

Dr Victoria Burrows

Dr Saima Ehsan

Dr Joe Kang

Dr Joni Tan

Dr Thomas Geh

Southampton

Dr Drew Maclean

Dr Sohini Chatterjee

Dr Bukola Ogunjinmi

Dr Marina Pearson
