# Supplementary material for: Evaluating current acute aortic syndrome pathways: Collaborative Acute Aortic Syndrome Project (CAASP)
Source: BJS Open. 2024 Sep 19;8(5):zrae096. doi: 10.1093/bjsopen/zrae096 (PMC11412149; doi:10.1093/bjsopen/zrae096)
Supplement: zrae096_Supplementary_Data [file zrae096_supplementary_data.zip › Supplementary_materials_2.docx]

**Supplemental information 2**

**Search strategy**

*Search on Hospital Radiology Information System (RIS).*

*Time Period: 01/01/2018 to 01/06/2021*

*Modality: CT Aorta*

*RIS Search Keywords (within clinical information section and main radiology report):*

*‘aortic syndrome’, ‘aortic dissection’, ‘penetrating ulcer’, ‘dissection flap’, ‘intramural haematoma’*
